# Impulsivity in patients with obsessive-compulsive disorder: exploring the mediating effect of cognitive emotion regulation strategies and depressive symptoms

**DOI:** 10.3389/fpsyt.2025.1668538

**Published:** 2025-11-14

**Authors:** Gang Ye, Meiling Chen, Liangjun Lin, Jia Li, Qichun Liu, Yanting Zhang, Zhen Tang, Ruihua Hou, Xiangdong Du

**Affiliations:** 1 Soochow University, Suzhou, China; 2 Suzhou Guangji Hospital, Soochow University Affiliated Guangji Hospital, Suzhou, China; 3 Clinical and Experimental Sciences, Faculty of Medicine, University of Southampton, Southampton, United Kingdom

**Keywords:** obsessive–compulsive disorder, impulsivity, cognitive emotion regulation, depressive symptoms, mediating effect

## Abstract

**Background:**

The underlying mechanism of impulsivity in obsessive-compulsive disorder (OCD) patients is complex and still unclear. Previous studies have not thoroughly explored whether impulsivity in OCD patients is a result of the obsessive-compulsive symptoms themselves or other contributing factors. This study aimed to explore whether cognitive emotion regulation strategies and depressive symptoms mediate the relationship between the severity of obsessive-compulsive symptoms and impulsivity in a clinical population with OCD.

**Methods:**

This was a case-control study that recruited 65 OCD patients (male/female=31/34) and 65 healthy controls (male/female =23/42), matched for age, gender, and education level. Demographic and clinical data were collected, and the Yale-Brown Obsessive Compulsive Scale (Y-BOCS), Beck Depression Inventory-II (BDI-II), Barratt Impulsiveness Scale-11 (BIS-11) and Cognitive Emotion Regulation Questionnaire (CERQ) were adopted.

**Results:**

OCD patients scored higher on BIS-11 attentional and non-planning impulsiveness and total scores (all *p* < 0.05). On CERQ, OCD patients showed elevated maladaptive strategies (self-blame, rumination, catastrophizing, blaming others) and reduced adaptive strategies (positive reappraisal) (all *p* < 0.05). Attentional impulsiveness positively correlated with OCD severity, depression, and maladaptive strategies (all *p* < 0.05). Non-planning impulsiveness and BIS-11 total scores positively correlated with depression and negatively with adaptive strategies (all *p* < 0.05). After adjusting for age, gender, depression level, there was only a significant negative correlation between BIS-11 non-planning impulsiveness and CERQ maladaptive strategies (*r* = -0.28, *p* < 0.05). Mediation analysis revealed significant indirect effects of OCD severity on impulsivity via adaptive strategies/depression (*β* = 0.13, 95% CI: 0.03~0.24, *p* = 0.012) and via maladaptive strategies/depression (*β* = 0.09, 95% CI: 0.00~0.23, *p* = 0.042), but no significant direct or total effects.

**Conclusions:**

OCD symptom severity indirectly influences impulsivity through emotion regulation strategies and depressive symptoms, highlighting the need to target these mediators in clinical interventions.

## 1 Introduction

Obsessive-compulsive disorder (OCD) is a psychiatric condition marked by the occurrence of obsessions and/or compulsive behaviors. Its lifetime prevalence in the general population ranges from 2% to 3% (1), contributing significantly to the economic strain on healthcare systems (2). The World Health Organization recognizes OCD as one of the ten most common disabling conditions (3).

As of now, the underlying pathogenesis of OCD is still not completely understood. The cognitive and behavioral model (4) is regarded as a traditional psychological framework which posits that compulsions persist and become excessive due to the immediate relief they provide from distress and the temporary alleviation of intrusive thoughts. Nevertheless, recent research has challenged this perspective, suggesting that OCD is linked to impulsivity, risky decision-making, and dysfunction within the reward system, aligning with a behavioral addiction viewpoint (5). From this angle, OCD is viewed similarly to addiction disorders, in which individuals develop a dependence on their compulsive behaviors because of the rewarding feelings that arise when these behaviors are performed correctly or when they reduce anxiety stemming from obsessions (6). This model emphasizes impulsivity as a critical factor in the psychological issues associated with OCD. Research indicates that OCD patients with higher impulsivity often display poorer insight, decreased resistance, a lower sense of control, and worse treatment outcomes (7).

Impulsivity refers to the inclination to respond quickly and without planning to either internal or external triggers disregarding possible negative outcomes (8). It is a multidimensional concept encompassing attentional, behavioral, and cognitive aspects (9). As a classic framework, the Barratt’s model deconstructs impulsivity into a multifaceted structure of three factors: attentional impulsiveness, motor impulsiveness, and non-planning impulsiveness, commonly measured using the Barratt Impulsiveness Scale-11 (BIS-11) (10). The UPPS-P model is another widely accepted model, which identifies five personality traits that are related to impulsivity: negative urgency, (lack of) premeditation, (lack of) perseverance, sensation seeking and positive urgency (11). Furthermore, based on biological foundations, the Reinforcement Sensitivity Theory (RST) suggests that personality dimensions related to impulsivity are mediated by the Behavioral Inhibition System (BIS) and the Behavioral Approach System (BAS) (12). Historically, compulsive and impulsive disorders have been conceptualized as opposites on a continuum, with compulsive disorders focused on avoiding harm and impulsive disorders aimed at seeking risk (13). Unlike impulsive behaviors, which are associated with a desire for immediate gratification, the compulsions in OCD themselves are not inherently pleasurable. Nonetheless, recent studies indicate that compulsivity and impulsivity are not diametrically opposed but instead function as independent dimensions, each playing distinct roles in psychiatric disorders such as those within the obsessive-compulsive spectrum, albeit to differing extents (14). Impulsive characteristics can develop over the extended progression of OCD, even without concurrent other impulse control disorders (15), and may affect treatment outcomes, adherence, and the overall prognosis of the disorder (16).

The underlying mechanisms of impulsivity in individuals with OCD are intricate and not yet fully understood. Previous research has not adequately addressed whether impulsivity in OCD patients originates from the obsessive-compulsive (OC) symptoms themselves or from other influences. Both compulsivity and impulsivity may reflect deficits in response inhibition or in top-down cognitive control. Most previous studies have focused on the shared neural pathways and the impaired function of specific neurotransmitters, including serotonin (5-HT) and dopamine (DA), within these pathways (13). These behaviors are likely influenced by interconnected but separate neural circuits involved in motivation and decision-making, which encompass the basal ganglia, limbic-cortical connections, and top-down control from the prefrontal cortex (17).

Studies suggest that emotion regulation—where individuals consciously or unconsciously modify their emotions to meet specific objectives—should be recognized as a transdiagnostic factor (18). The process model of Gross is one of the most influential models in the field, which includes five steps: selection of the situation, modification of the situation, deployment of attention, change of cognitions, and modulation of responses, describes the regulatory strategies used at different stages of emotion generation (19). Meanwhile, several scholars proposed other different theoretical models. For example, Baumeister’s self-regulatory strength model regards emotion regulation as a self-control behavior, explaining regulatory failures through resource depletion (20). Difficulties in emotion regulation involve various impairments, including difficulty controlling impulses (21). Deficits in emotional regulation have been correlated with both the onset and continuation of OCD (22) as well as impulsive disorders (23, 24). Currently, there are various self-report questionnaires designed to evaluate aspects of emotion regulation; however, many of these tools do not specifically or directly evaluate the cognitive processes involved in emotion regulation (25). Since impulsivity encompasses cognitive components (9), examining the cognitive strategies behind emotion regulation in impulsive individuals is crucial. The Cognitive Emotion Regulation Questionnaire (CERQ) was created to assess particular cognitive strategies for emotion regulation that individuals employ when faced with stressful life situations, which can more accurately quantify and assess emotion regulation problems.

Impulsivity has been seen as connected with depression (26) and depressive symptoms frequently co-occur with OC symptoms (27). Depressive symptoms may increase the severity of OC symptoms (28), which in turn are associated with higher levels of impulsivity (29). Therefore, OCD patients comorbid with depression may exhibit higher impulsivity. Additionally, impulsivity, OC symptoms and depression may share common risk factors. In terms of neurochemistry, dysfunction of the 5-HT system implicated in all three conditions (30–32). From neuroimaging aspect, as the core of OCD pathology, CSTC circuit dysfunction (1) is also related to depression (33) and impulsivity (34). Consequently, it is crucial to investigate how emotion regulation and depressive symptoms moderate impulsivity in this population.

This study investigated the mediating role of cognitive emotion regulation strategies and depressive symptoms in the relationship between OC symptom severity and impulsivity among individuals clinically diagnosed with OCD. Cognitive emotion regulation strategies involve mental processes triggered by events that evoke emotions, which intentionally or unintentionally seek to change the strength and/or quality of a person’s emotional response (35). We hypothesize that (i) patients with OCD tend to be more impulsive and employ more maladaptive and fewer adaptive cognitive emotion regulation strategies compared to healthy controls; (ii) cognitive emotion regulation strategies and depressive symptoms mediate the effect of OC symptom severity on impulsivity.

## 2 Material and methods

Given the complex and often paradoxical interrelationships between impulsivity, compulsivity, and negative affect observed in OCD, a more thorough investigation is warranted. Previous studies have frequently examined these constructs in isolation, unable to understand how these factors interact with each other. This study adopted a case-controlled cross-sectional design to investigate the differences in impulsivity and the use of cognitive emotion regulation strategies between OCD patients and healthy controls, and to explore the interrelationships between OC symptoms, cognitive emotion regulation, depression, and impulsivity through the mediation analysis.

### 2.1 Participants

The sample size estimation was performed using G*Power (version 3.1), a statistical analysis tool. We utilized a two-sided test with α=0.05, β=0.2 (80% power), a medium effect size, and equal sample sizes for both groups. According to Cohen’s recommendations (36), a medium effect size is between 0.5 and 0.8. Therefore, the range of sample sizes for each group was calculated to be 26-64. Ultimately, we included 65 participants in each group.

OCD patient group: Sixty-five patients (male/female=31/34) with OCD were recruited from both the outpatient clinic and inpatient unit at Suzhou Guangji Hospital. All participants met the following inclusion criteria: (i) aged between 18 and 65 years; (ii) diagnosed with OCD according to the Statistical Manual of Mental Disorders, Fifth Edition (DSM-5); (iii) Yale-Brown Obsessive Compulsive Scale (Y-BOCS) score≥8; (iv) had 6 or more years of education; and (v) ability to engage in the clinical assessment. Exclusion criteria included any current or past psychosis, major depressive disorder, bipolar disorder, substance abuse, active suicide ideation and/or suicide attempt.

Healthy control group: Sixty-five healthy participants (male/female=23/42) were recruited through advertisements in local communities in Suzhou. These individuals were healthy volunteers aged from 18 to 65 years.

All participants received a screening interview to identify any comorbid mental disorders using the Mini International Neuropsychiatric Interview (MINI). This study received approval from the Clinical Research Ethics Committee of Suzhou Guangji Hospital (No. 2021-021). All procedures conducted in this study were carried out strictly followed with the Declaration of Helsinki. Written informed consent was obtained from all participants prior to their involvement in the study.

### 2.2 Clinical measurements

#### 2.2.1 Yale-Brown Obsessive Compulsive Scale (Y-BOCS)

The Y-BOCS is the most commonly utilized clinician-rated instrument for measuring OCD symptom severity. It consists of 10 core items, each scored from 0 (no symptoms) to 4 (severe symptoms), with a total score ranging from 0 to 40, which 8–15 represent mild symptoms, 16–23 relate to moderate symptoms, 24–31 suggest severe symptoms, and 32–40 imply extreme symptoms (37). The first five items assess obsessions, while the last five evaluate compulsions. The Chinese version of the Y-BOCS demonstrates convincing content validity and construction validity, good internal consistency (Cronbach’s alpha=0.75), high interrater reliability (ICC = 0.82), and reliable test-retest reliability (ICC = 0.75) (38).

#### 2.2.2 Beck Depression Inventory-II (BDI-II)

The BDI-II is a structured self-reported questionnaire designed to measure the level of individual distress caused by various depressive symptoms. It includes 21 items, each scored from 0 to 3. The Chinese version of the BDI-II contains two factors: the somatic-affective factor and the cognitive factor. It has strong internal consistency (Cronbach’s alpha=0.94) and moderate test-retest reliability (ICC = 0.55) (39).

#### 2.2.3 Barrant Impulsiveness Scale-11 (BIS-11)

The BIS-11 is the most widely utilized self-report tool to assess impulsiveness (40), which consists of 30 items rated on a Likert scale, with scores ranging from 1 point (never) to 4 points (very frequently). Our study used the 26-item Chinese version of the BIS-11, derived from its initial English-language version. This instrument includes 6 items measuring attentional impulsiveness, 10 items assessing motor impulsiveness, and 10 items evaluating non-planning impulsiveness. Eleven of the 26 items are reverse-scored. According to previous studies in Chinese college students and community residents (41), the Chinese version of the BIS-11 has good internal consistency (Cronbach’s alpha=0.759) and strong test-retest reliability (ICC = 0.853).

#### 2.2.4 Cognitive Emotion Regulation Questionnaire (CERQ)

The CERQ consists of 36 items, employs a 5-point Likert scale and covers nine subscales, including four maladaptive strategies (self-blame, rumination, catastrophizing, and blaming others) and five adaptive strategies (acceptance, positive refocusing, refocusing on planning, positive reappraisal, and putting into perspective). The Chinese version of the CERQ has shown acceptable internal consistency (Cronbach’s alpha=0.83) and test-retest reliability (ICC = 0.64) (42).

### 2.3 Statistical analysis

Data analysis was performed via the Statistical Package for Social Sciences (SPSS version 22). Through Q-Q plots and calculations of skewness and kurtosis, the data from the OCD and the control group conformed to an approximate normal distribution. The equality of variance was assessed by Levene’s test to determine the appropriate statistical results. Due to the small sample size, in order to retain as many participants as possible, subjects with outliers were not excluded. We used the mean instead of outliers for correction. Descriptive statistics were used to characterize the sample’s demographic profile and Y-BOCS, BDI-II, BIS-11 and CERQ scores. Differences in demographic profiles and BIS-11 and CERQ scores between the OCD and control groups were compared with chi-square tests for categorical variables and independent *t*-tests for continuous variables. ANCOVA was used to compare the BIS-11 and CERQ scores among OCD patients with different severity levels, including medication status, psychotherapy exposure, and inpatient/outpatient recruitment setting as covariates. Bonferroni corrections were applied in group comparisons. Pearson’s correlation analysis examined relationships among the Y-BOCS, BDI-II, CERQ, and BIS-11 scores. The mediation analysis were conducted using Amos (version 22.0). The variables used in the model were as follows: Y-BOCS total score as the independent variable, impulsivity as the dependent variable, cognitive emotion regulation strategies and depressive symptoms as mediating variables. All *p*-values were 2-tailed at a significance level of <0.05.

## 3 Results

### 3.1 Participant characteristics and clinical profiles

**Table 1** shows the demographic and clinical characteristics of OCD patients and controls. There were no significant differences between the two groups in terms of age (*t* = -1.97, *p* = 0.052), gender (*χ2* = 2.03, *p* = 0.154) and education level (*t* = -1.86, *p* = 0.065).

**Table 1:** Demographic and clinical characteristics of OCD patients and controls.

Variables	OCD group (n=65)	Control group (n=65)	*t* or *χ^2^*	*p*
Age (years, mean ± SD)	29.29 ± 6.71	31.92 ± 8.45	-1.97	0.052
Gender (male/female)	31/34	23/42	2.03	0.154
Education level (years, mean ± SD)	14.45 ± 2.73	15.32 ± 2.63	-1.86	0.065
Age of onset (years, mean ± SD)	21.13 ± 7.01			
Disease duration (years, mean ± SD)	8.18 ± 5.18			
Duration of this episode (months, mean ± SD)	6.36 ± 5.00			
Medical treatment (yes/no)	39/26			
Psychotherapy (yes/no)	10/55			
Recruitment setting (outpatient/inpatient)	29/36			
Y-BOCS-Obsessions	11.82 ± 3.82			
Y-BOCS-Compulsions	11.05 ± 3.68			
Y-BOCS-Total	22.86 ± 6.65			
BDI-II	16.98 ± 11.00			

OCD, Obsessive-Compulsive Disorder; Y-BOCS, Yale-Brown Obsessive Compulsive Scale; BDI-II, Beck Depression Inventory-II.

### 3.2 Comparisons of BIS-11 and CERQ scores among OCD patients with different severity levels and controls

**Tables 2** and **3** shows comparisons of questionnaire measures of impulsivity and cognitive emotion regulation strategies. For the comparison between OCD patients and controls (**Table 2**), OCD patients scored higher than controls on attentional impulsiveness (*t* = 5.17, *p* < 0.001), non-planning impulsiveness (*t* = 2.13, *p* = 0.035) subscales and total score (*t* = 3.18, *p* = 0.002) on the BIS-11. On the CERQ, OCD patients scored higher than controls on self-blame (*t* = 2.24, *p* = 0.027), rumination (*t* = 4.94, *p* < 0.001), catastrophizing (*t* = 6.15, *p* < 0.001), blaming others (*t* = 2.90, *p* = 0.004) strategies and total score of maladaptive strategies subscales (*t* = 5.86, *p* < 0.001) meanwhile lower than controls on positive reappraisal (*t* = -5.85, *p* < 0.001) and total score of adaptive strategies subscales (*t* = -2.74, *p* = 0.007). There was no significant difference between the two groups of participants on the motor impulsiveness (*t* = 0.80, *p* = 0.425) subscale. There were no significant differences between the two groups of participants on the acceptance (*t* = -1.26, *p* = 0.211), positive refocusing (*t* = -1.91, *p* = 0.058), refocusing on planning (*t* = -0.47, *p* = 0.640), and putting into perspective (*t* = 0.83, *p* = 0.407) subscales. According to **Table 3**, there were significant differences in attentional impulsiveness (*F* = 6.74, *p* = 0.002, partial η²=0.186) and BIS-11 total score (*F* = 3.65, *p* = 0.032, partial η²=0.110) among OCD patients with different severity levels. Meanwhile, putting into perspective (*F* = 10.04, *p* < 0.001, partial η²=0.254) and catastrophizing (*F* = 7.58, *p* = 0.001, partial η²=0.204) subscales of the CERQ also had significant results.

**Table 2:** Comparisons of BIS-11 and CERQ scores between OCD patients and controls.

Variables	OCD group (n=65)	Control group (n=65)	*t*	*p*
BIS-11-Attentional	14.02 ± 3.12	11.45 ± 2.51	5.17	<0.001^**^
BIS-11-Motor	18.23 ± 4.12	17.72 ± 3.08	0.80	0.425
BIS-11-Non-planning	21.77 ± 4.73	20.09 ± 4.23	2.13	0.035^*^
BIS-11-Total	54.02 ± 9.08	49.39 ± 7.35	3.18	0.002^**^
CERQ-Self-blame	12.49 ± 2.75	11.52 ± 2.17	2.24	0.027^*^
CERQ-Acceptance	13.52 ± 2.91	14.18 ± 3.09	-1.26	0.211
CERQ-Rumination	12.74 ± 3.52	9.82 ± 3.22	4.94	<0.001^**^
CERQ-Positive refocusing,	10.75 ± 2.96	11.71 ± 2.73	-1.91	0.058
CERQ-Refocusing on planning	14.09 ± 3.41	14.35 ± 2.94	-0.47	0.640
CERQ-Positive reappraisal	11.78 ± 3.46	14.98 ± 2.74	-5.85	<0.001^**^
CERQ-Putting into perspective	10.52 ± 2.41	10.15 ± 2.65	0.83	0.407
CERQ-Catastrophizing	10.97 ± 3.61	7.34 ± 3.09	6.15	<0.001^**^
CERQ-Blaming others	10.15 ± 3.42	8.42 ± 3.41	2.90	0.004^**^
CERQ-Adaptive strategies	60.68 ± 10.18	65.38 ± 9.41	-2.74	0.007^**^
CERQ-Maladaptive strategies	46.35 ± 9.10	37.09 ± 8.94	5.86	<0.001^**^

OCD, Obsessive-Compulsive Disorder; BIS-11, Barratt Impulsiveness Scale-11; CERQ, Cognitive Emotion Regulation Questionnaire. ^*^p < 0.05; ^**^p < 0.01.

**Table 3:** Comparisons of BIS-11 and CERQ scores among OCD patients with different severity levels.

Variables	Mild OCD group^1^ (n=10)	Moderate OCD group^2^ (n=25)	Severe OCD group^3^ (n=30)	*F*	*p*	partial η²
BIS-11-Attentional	11.00 ± 3.53	14.48 ± 3.00	14.63 ± 2.55	6.74	0.002^**a^	0.186
BIS-11-Motor	17.30 ± 4.90	18.88 ± 4.06	18.00 ± 3.97	0.46	0.635	0.015
BIS-11-Non-planning	18.40 ± 4.93	22.12 ± 4.98	22.60 ± 4.07	2.54	0.087	0.079
BIS-11-Total	46.70 ± 11.43	55.48 ± 8.88	55.23 ± 7.37	3.65	0.032^*b^	0.110
CERQ-Self-blame	11.40 ± 2.22	12.48 ± 2.71	12.87 ± 2.91	1.00	0.372	0.033
CERQ-Acceptance	13.30 ± 2.11	14.44 ± 2.97	12.83 ± 2.96	1.90	0.159	0.060
CERQ-Rumination	10.80 ± 2.62	12.84 ± 3.46	13.30 ± 3.71	1.71	0.190	0.055
CERQ-Positive refocusing,	10.20 ± 3.43	11.40 ± 2.43	10.40 ± 3.19	0.83	0.443	0.027
CERQ-Refocusing on planning	14.20 ± 2.90	14.20 ± 2.74	13.97 ± 4.10	0.03	0.973	0.001
CERQ-Positive reappraisal	13.00 ± 3.65	11.88 ± 3.15	11.30 ± 3.64	0.86	0.429	0.028
CERQ-Putting into perspective	8.80 ± 2.20	11.72 ± 1.84	10.10 ± 2.45	10.04	<0.001^**c^	0.254
CERQ-Catastrophizing	8.60 ± 3.06	12.76 ± 3.10	10.27 ± 3.57	7.58	0.001^**d^	0.204
CERQ-Blaming others	10.80 ± 2.53	11.12 ± 3.49	9.13 ± 3.42	2.74	0.073	0.085
CERQ-Adaptive strategies	59.50 ± 8.10	63.64 ± 8.29	58.60 ± 11.78	1.93	0.155	0.061
CERQ-Maladaptive strategies	41.60 ± 5.95	49.20 ± 10.07	45.57 ± 8.50	3.01	0.057	0.093

OCD, Obsessive-Compulsive Disorder; BIS-11, Barratt Impulsiveness Scale-11; CERQ, Cognitive Emotion Regulation Questionnaire. * *p* < 0.05; ** *p* < 0.01.

All *post-hoc* analysis were done using Bonferroni:

^a1^*vs.*^2^*p* = 0.006^**^, ^1^*vs.*^3^*p* = 0.002^**^, ^2^*vs.*^3^*p* = 1.000.

^b1^*vs.*^2^*p* = 0.048^*^, ^1^*vs.*^3^*p* = 0.040^*^, ^2^*vs.*^3^*p* = 1.000.

^c1^*vs.*^2^*p* < 0.001^**^, ^1^*vs.*^3^*p* = 0.156, ^2^*vs.*^3^*p* = 0.008^**^.

^d1^*vs.*^2^*p* = 0.002^**^, ^1^*vs.*^3^*p* = 0.238, ^2^*vs.*^3^*p* = 0.028^*^.

### 3.3 Associations among scores of Y-BOCS, BDI-II, CERQ and BIS-11 in patientswith OCD

**Table 4** shows the relationship between questionnaire measures of OC symptom severity, depression level, cognitive emotion regulation strategies and impulsivity. Significant positive correlations were found among obsessions (*r* = 0.26, *p* < 0.05), compulsions (*r* = 0.29, *p* < 0.05), Y-BOCS total score (*r* = 0.31, *p* < 0.05), BDI-II score (*r* = 0.37, *p* < 0.01), CERQ maladaptive strategies (*r* = 0.33, *p* < 0.05) and the BIS-11 attentional impulsiveness. Additionally, BIS-11 non-planning impulsiveness (*r* = 0.38, *p* < 0.01) and total BIS-11 score (*r* = 0.33, *p* < 0.01) exhibited significant positive correlations with the BDI-II score. BIS-11 non-planning impulsiveness (*r* = -0.41, *p* < 0.01) and total BIS-11 score (*r* = -0.25, *p* < 0.05) also exhibited significant negative correlations with CERQ adaptive strategies. After adjusting for age, gender, depression level, there was only a significant negative correlation between BIS-11 non-planning impulsiveness and CERQ maladaptive strategies (*r* = -0.28, *p* < 0.05).

**Table 4:** Associations among scores of Y-BOCS, BDI-II, CERQ and BIS-11 in patients with OCD.

Variables	BIS-11- Attentional	BIS-11- Motor	BIS-11- Non-planning	BIS-11- Total
Y-BOCS-Obsessions	0.26^*^	0.03	0.23	0.22
Y-BOCS-Compulsions	0.29^*^	0.01	0.11	0.16
Y-BOCS-Total	0.31^*^	0.02	0.20	0.22
BDI-II	0.37^**^	0.01	0.38^**^	0.33^**^
CERQ-Adaptive strategies	0.11	-0.16	-0.41^**^	-0.25^*^
CERQ-Maladaptive strategies	0.33^*^	0.04	-0.10	0.08

Y-BOCS, Yale-Brown Obsessive Compulsive Scale; BDI-II, Beck Depression Inventory-II; BIS-11, Barratt Impulsiveness Scale-11; CERQ, Cognitive Emotion Regulation Questionnaire.

^*^p < 0.05; ^**^p < 0.01.

### 3.4 Chained mediating analysis

**Figure 1:**
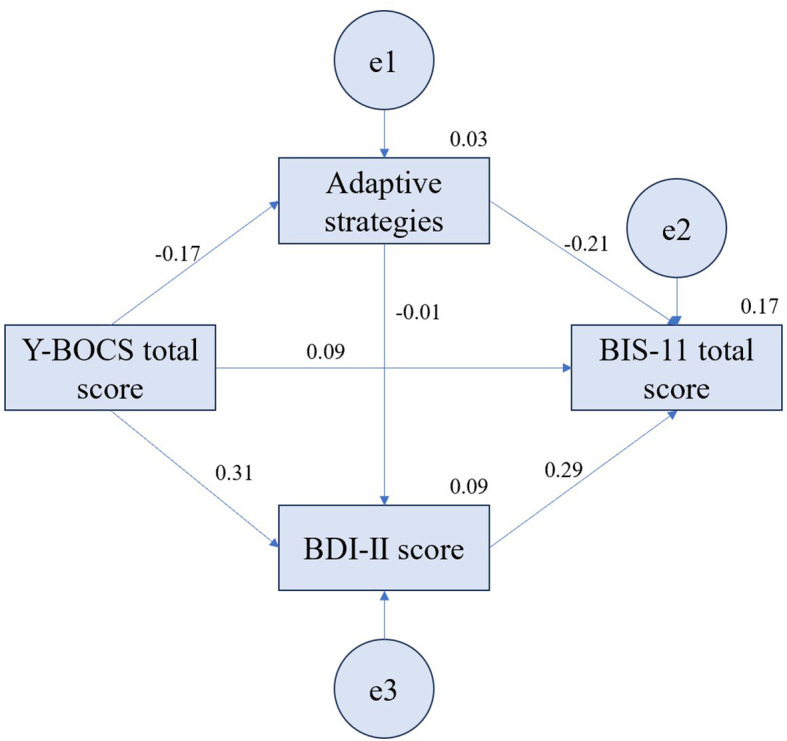
Model 1: The model of mediating role of adaptive strategies and BDI-II score between Y-BOCS total score and BIS-11 total score in OCD patients.

**Figure 1** shows the model of mediating role of adaptive strategies and BDI-II score between Y-BOCS total score and BIS-11 total score in OCD patients (Model 1). As a saturated model, the indices of goodness-of-fit of the model were as follows, *χ2* = 0.000, *df* = 0, CMIN = 0.000, GFI = 1.000, CFI = 1.000, NFI = 1.000, IFI = 1.000, RMR = 0.000, SRMR = 0.000. **Table 5** shows that the Y-BOCS total score has a significant positive predictive effect on BDI-II score (*p* < 0.05), and BDI-II score has a significant positive predictive effect on BIS-11 total score (*p* < 0.05). The theoretical model was tested by estimating the 95% confidence intervals (CI) for mediation effects with 5000 bootstrap samples. If the 95% CI did not cross 0, it meant that the effect was significant. **Table 6** shows the results of the standardized effects and 95% CI for direct and indirect associations of adaptive strategies and BDI-II score between Y-BOCS total score and BIS-11 total score in OCD patients via bootstrap. The bootstrap test showed that the indirect effect was significant (*β* = 0.13, 95% CI: 0.03~0.24, *p* = 0.012) but the direct (*β* = 0.09, 95% CI: -0.18~0.36, *p* = 0.490) and total effects (*β* = 0.22, 95% CI: -0.09~0.46, *p* = 0.156) were not significant. Regarding the effect of adaptive strategies on BIS-11 total score of Model 1 (**Supplementary Table 1**), it showed that the all the paths were not significant. For the effect of BDI-II score on BIS-11 total score of Model 1 (**Supplementary Table 2**), there was only direct effect which was significant (*β* = 0.29, 95% CI: 0.05~0.50, *p* = 0.023), but no indirect effect. The models of mediating role of adaptive strategies and BDI-II score between Y-BOCS total score and BIS-11 subscale scores were also tested. For the effect of Y-BOCS total score on BIS-11 non-planning impulsiveness score, the bootstrap test showed that the indirect effect was significant (*β* = 0.17, 95% CI: 0.05~0.30, *p* = 0.010) but the direct (*β* = 0.02, 95% CI: -0.22~0.28, *p* = 0.811) and total effects (*β* = 0.20, 95% CI: -0.09~0.44, *p* = 0.174) were not significant. The direct (*β* = 0.24, 95% CI: 0.02~0.46, *p* = 0.039) and total effects (*β* = 0.31, 95% CI: 0.07~0.50, *p* = 0.015) of Y-BOCS total score on BIS-11 attentional impulsiveness score were significant but the indirect effect (*β* = 0.07, 95% CI: -0.06~0.20, *p* = 0.300) was not significant (**Supplementary Tables 3**-**6**). Additionally, all paths of mediating effects of Y-BOCS total score on BIS-11 motor impulsiveness score were nonsignificant.

**Table 5:** Model 1: mediating effects of adaptive strategies and BDI-II score between Y-BOCS total score and BIS-11 total score in OCD patients.

Paths	*β*	SE	95% CI	*p*
Y-BOCS total score→BIS-11 total score	0.09	0.14	-0.18~0.36	0.490
Y-BOCS total score→Adaptive strategies	-0.17	0.14	-0.41~0.13	0.255
Y-BOCS total score→BDI-II score	0.31	0.12	0.04~0.52	0.023^*^
Adaptive strategies→BDI-II score	-0.01	0.16	-0.30~0.30	0.977
Adaptive strategies→BIS-11 total score	-0.21	0.13	-0.48~0.06	0.113
BDI-II score→BIS-11 total score	0.29	0.12	0.05~0.50	0.023^*^

Y-BOCS, Yale-Brown Obsessive Compulsive Scale; BDI-II, Beck Depression Inventory-II;

BIS-11, Barratt Impulsiveness scale-11. ^*^p < 0.05.

**Table 6:** The paths and effect analysis of Y-BOCS total score→BIS-11 total score of model 1.

Effect	*β*	SE	95% CI	*p*
Direct effect	0.09	0.14	-0.18~0.36	0.490
Indirect effect	0.13	0.05	0.03~0.24	0.012^*^
Total effect	0.22	0.14	-0.09~0.46	0.156

^*^p < 0.05.

**Figure 2:**
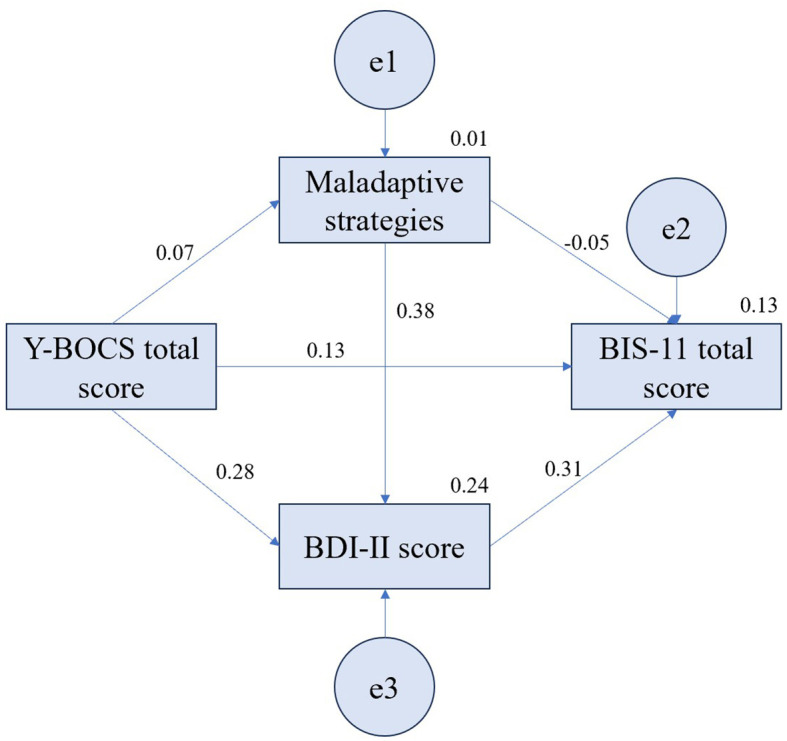
Model 2: The model of mediating role of maladaptive strategies and BDI-II score between Y-BOCS total score and BIS-11 total score in OCD patients.

**Figure 2** shows the model of mediating role of maladaptive strategies and BDI-II score between Y-BOCS total score and BIS-11 total score in OCD patients (Model 2). The indices of goodness-of-fit of the model were as follows, *χ2* = 0.000, *df* = 0, CMIN = 0.000, GFI = 1.000, CFI = 1.000, NFI = 1.000, IFI = 1.000, RMR = 0.000, SRMR = 0.000. **Table 7** shows that the Y-BOCS total score has a significant positive predictive effect on BDI-II score (*p* < 0.05), maladaptive strategies have a significant positive predictive effect on BDI-II score (*p* < 0.01), and BDI-II score has a borderline significant positive predictive effect on BIS-11 total score (*p* = 0.050).  **Table 8** shows the results of the standardized effects and 95% CI for direct and indirect associations of maladaptive strategies and BDI-II score between Y-BOCS total score and BIS-11 total score in OCD patients via bootstrap. The bootstrap test showed that the indirect effect was significant (*β* = 0.09, 95% CI: 0.00~0.23, *p* = 0.042) but the direct (*β* = 0.13, 95% CI: -0.17~0.40, *p* = 0.383) and total effects (*β* = 0.22, 95% CI: -0.09~0.46, *p* = 0.156) were not significant. In regard to the effect of maladaptive strategies on BIS-11 total score of Model 2 (**Supplementary Table 7**), the bootstrap test showed that both direct (*β* = -0.05, 95% CI: -0.33~0.25, *p* = 0.689) and total effects (*β* = 0.07, 95% CI: -0.20~0.33, *p* = 0.669) were nonsignificant, but the indirect effect was significant (*β* = 0.12, 95% CI: 0.02~0.29, *p* = 0.025). Regarding the effect of BDI-II score on BIS-11 total score of Model 2 (**Supplementary Table 8**), the direct effect was also equal to total effect, which was borderline significant (*β* = 0.31, 95% CI: 0.00~0.57, *p* = 0.050), while there was no indirect effect. Further modeling on BIS-11 subscales shows that the indirect effect of Y-BOCS total score on BIS-11 non-planning impulsiveness score was significant (*β* = 0.13, 95% CI: 0.02~0.27, *p* = 0.022) but the direct (*β* = 0.07, 95% CI: -0.21~0.34, *p* = 0.587) and total effects (*β* = 0.20, 95% CI: -0.09~0.44, *p* = 0.174) were not significant. The direct (*β* = 0.23, 95% CI: 0.02~0.43, *p* = 0.034) and total effects (*β* = 0.31, 95% CI: 0.07~0.50, *p* = 0.015) of Y-BOCS total score on BIS-11 attentional impulsiveness score were significant, but the indirect effect (*β* = 0.08, 95% CI: -0.03~0.21, *p* = 0.140) was not significant (**Supplementary Tables 9**–**12**). Besides, the mediating effects of Y-BOCS total score on BIS-11 motor impulsiveness score were not significant in all paths either.

**Table 7:** Model 2: mediating effects of maladaptive strategies and BDI-II score between Y-BOCS total score and BIS-11 total score in OCD patients.

Paths	*β*	SE	95% CI	*p*
Y-BOCS total score→BIS-11 total score	0.13	0.15	-0.17~0.40	0.383
Y-BOCS total score→Maladaptive strategies	0.07	0.13	-0.18~0.31	0.600
Y-BOCS total score→BDI-II score	0.28	0.11	0.05~0.49	0.016^*^
Maladaptive strategies→BDI-II score	0.38	0.12	0.13~0.58	0.002^**^
Maladaptive strategies→BIS-11 total score	-0.05	0.15	-0.33~0.25	0.689
BDI-II score→BIS-11 total score	0.31	0.15	0.00~0.57	0.050

Y-BOCS, Yale-Brown Obsessive Compulsive Scale; BDI-II, Beck Depression Inventory-II; BIS-11, Barratt Impulsiveness Scale-11. ^*^p < 0.05, **p < 0.01.

**Table 8:** The paths and effect analysis of Y-BOCS total score→BIS-11 total score of model 2.

Effect	*β*	SE	95% CI	*P*
Direct effect	0.13	0.15	-0.17~0.40	0.383
Indirect effect	0.09	0.06	0.00~0.23	0.042^*^
Total effect	0.22	0.14	-0.09~0.46	0.156

^*^p < 0.05.

## 4 Discussion

To the best of our knowledge, this is the first study to explore the mediating effect of cognitive emotion regulation strategies and depressive symptoms between OC symptom severity and impulsivity in a clinical OCD population. Our study found that (i) OCD patients demonstrated increased self-reported attentional, non-planning impulsiveness and total impulsiveness scores; (ii) OCD patients used more maladaptive and less adaptive cognitive emotion regulation strategies; (iii) In unadjusted analysis, OCD symptom severity, depressive symptoms and cognitive emotion regulation strategies were associated with different facets of impulsivity. However, most associations did not remain statistically significant after adjustment for confounding factors; (iv) OC symptom severity only had an indirect effect on impulsivity mediated by cognitive emotion regulation strategies and depressive symptoms.

Traditional perspectives generally regard compulsivity and impulsivity as entirely opposing concepts; however, more current interpretations indicate that there may be considerable overlap between the two, with both being recognized as behavioral traits linked to OCD (43). In our study, OCD patients showed increased scores on two (attentional and non-planning impulsiveness) out of three BIS-11 subscales and the total impulsiveness score. This result supports the idea that cognitive impulsivity is especially common in OCD patients (5). Previous studies have shown that OCD patients tend to have higher scores on the attentional subscale and overall on the BIS-11, although there are some controversies regarding the non-planning and motor subscales (44). The increased attentional impulsiveness may be indicative of the intrusive and uncontrollable thoughts experienced by these individuals (45). Moreover, previous research has shown that symptoms of childhood attention deficit hyperactivity disorder (ADHD) are more prevalent among patients with OCD, and multiple findings have also identified potential etiological and pathophysiological links between the two conditions (29).

Although difficulties in regulating emotions and the reliance on maladaptive strategies to cope with distress are recognized as central to the psychopathology of OCD (46), to our knowledge, cognitive emotion regulation has not yet been fully studied in patients with OCD. Our study indicated that OCD patients employed more maladaptive and less adaptive cognitive strategies. This is consistent with previous research that emphasizes emotion regulation deficits in individuals with OCD, especially the common use of maladaptive strategies and a diminished use of reappraisal techniques (47). Additionally, studies conducted with nonclinical populations have indicated that difficulties in emotion regulation—such as self-blame, rumination, catastrophizing, and blaming others—are associated with an increase in OC symptoms (22, 48, 49). Cognitive models of OCD propose that the way individuals with OCD evaluate intrusive thoughts leads to significant distress and prompts efforts to suppress these thoughts, along with engaging in ritualistic behaviors (22). The Obsessive Compulsive Cognitions Working Group proposed three key types of dysfunctional beliefs that contribute to the symptoms of OCD: (i) overestimation of threat and heightened sense of responsibility; (ii) beliefs regarding the importance of intrusive thoughts and the necessity to control them; and (iii) perfectionism coupled with an intolerance of uncertainty (50). The concept of overestimation of threats involves a tendency to catastrophize, while inflated responsibility often manifests as self-blame. Rumination can cause OCD patients to become addicted to their feelings and thoughts about things they have experienced. Continuously recalling their feelings about things they have experienced further reinforces their implicit compulsive behavior (51). Emotional regulation is believed to result from a complex interplay between automatic bottom-up appraisals of stimuli in lower brain regions, such as the amygdala, and top-down cognitive appraisals from higher brain regions, including the dorsolateral and dorsomedial prefrontal cortices (52). Neuroimaging studies in individuals with OCD have revealed deficiencies in brain areas associated with these ventral-dorsal circuits, suggesting that the failure of emotional regulation in patients with OCD may stem from impairments in dorsal control functions and/or excessive activation in the ventral system (52).

Gratz and Roemer’s model of emotional dysregulation suggests that effective emotion regulation involves the capacity to control impulses and coordinate actions with intended objectives when faced with distressing situations (21). Research has suggested that impaired emotional regulation may act as a common factor underlying participation in diverse impulsive actions (53). In our study, we found that cognitive emotion regulation strategies might influence different facets of impulsivity in OCD patients. Overall, individuals with OCD who used more maladaptive and fewer adaptive strategies, as assessed by the CERQ, demonstrated greater levels of impulsivity, especially in terms of attentional and non-planning impulsiveness. The theoretical model of emotional regulation developed by Gross’s (19) explains that emotion regulation includes both antecedent-focused strategies, such as selecting and modifying situations and deploying attention, as well as response-focused strategies, which involve cognitive change and modulating responses. Struggles with impulse control indicate a person’s inability to regulate their behavior in reaction to distressing emotions (54). A recent systematic review indicated that difficulties with impulse control could be viewed as a construct of emotion regulation deficits in patients with OCD (54). Notably, our study found that not all aspects of impulsivity were linked to emotion regulation. Attentional impulsiveness is characterized by issues with attention and cognitive instability, whereas non-planning impulsiveness is associated with self-control and cognitive complexity (41), both encompassing cognitive aspects of impulsivity. Because the CERQ mainly evaluates cognitive styles connected to emotional regulation, it is likely to have a stronger association with attentional and non-planning impulsiveness. However, most associations did not remain statistically significant after adjustment for confounding factors, indicating that they may be preliminary and require further validation in larger samples.

Several studies indicated that individuals with OCD reported significantly higher levels of impulsivity, which were largely influenced by the severity of their OC symptoms (29). Nonetheless, earlier research has not fully investigated whether impulsivity in individuals with OCD is a result of the OC symptoms or other contributing factors. Our study found that the severity of OC symptoms showed no significant direct effect on impulsivity, but it could indirectly affect impulsivity through the mediating roles of cognitive emotion regulation strategies and depressive symptoms. This suggests that the severity of OC symptom is not directly linked to impulsivity. Research on impulsivity often suggests that deficiencies in inhibitory control are the main cause of impulsive behavior (55). While response inhibition impairments are regarded as one of the endophenotypic markers for OCD, research outcomes regarding this area in OCD patients remain inconclusive. A body of evidence has revealed that individuals with OCD exhibit impaired response inhibition, while others have observed no significant differences when compared to healthy controls (56). The inconsistent results have been attributed to various factors, including the high prevalence of comorbidity with depression (56). The connection between the OC symptom severity and depression has already been thoroughly studied. Besiroglu et al. discovered that in patients with OCD, more severe obsessions were associated with depressive symptoms and could serve as predictors for a depression diagnosis (57). Altintaş et al. found that the severity of both obsession and compulsion is positively correlated with the severity of depression (58). The comorbidity of OCD and depression can be seen as stemming from overlapping genetic, physiological, psychological, and social factors (27). Cognitive strategies for managing emotions play a pivotal role in psychological functioning, and their deficiencies are believed to correlate with depression symptoms (59). Individuals prone to depression inclined to depend on dysfunction strategies more often than on adaptive approaches (59). Patients with OCD can indirectly impact their impulsivity by employing cognitive emotion regulation strategies to modify the severity of their depressive symptoms.

This research has certain limitations. Firstly, the cross-sectional design of our study fundamentally constrains causal inference. Secondly, our measures of impulsivity were derived from self-report questionnaires, which assess the subjective perception of one’s impulsive traits. These lack the objective performance data provided by neurocognitive tasks. Therefore, we were unable to capture the specific behavioral and cognitive mechanisms of impulsivity, potentially creating a disconnection between reported impulsivity and its actual behavioral manifestation. Thirdly, this study relies on a single model of impulsivity, as measured by the BIS-11. While this instrument is widely used and has demonstrated value, other conceptual frameworks, such as the BIS/BAS (Behavioral Inhibition System/Behavioral Approach System) model, might capture distinct yet relevant aspects of impulsive traits and motivational tendencies that were not assessed here. It is noteworthy, however, that our findings are consistent with other recent researches (60). Fourthly, participants were not assessed for ADHD symptoms in this study. Impulsivity is a multidimensional construct that manifests differently across psychiatric disorders. If participants were comorbid ADHD symptoms, the measured “impulsivity” might reflect a heterogeneous mixture of traits. Therefore, the results might not reflect a pure OCD-specific impulsive trait but rather a fusion of features associated with ADHD, thereby confounding theoretical conclusions. Fifthly, the limited sample size may reduce the generalizability of the results. Larger studies are needed to validate these findings. Lastly, the SEM model in this study is a saturated model. Although the saturated model could not effectively test the goodness of fit, the SEM used in this study was mainly for path analysis, and our focus was on parameter estimates rather than overall model fit.

Future research should employ longitudinal designs to establish causality and explore the developmental trajectories of these relationships over time. In order to gain a more comprehensive and objective understanding of impulsivity, future work should adopt multimodal assessment approaches such as neurocognitive tests. What’s more, our findings indicate that there is a compelling need for intervention studies that specifically target maladaptive emotion regulation strategies to reduce impulsivity in patients with OCD. Cognitive therapy targeting emotional regulation could serve as a promising approach to managing impulsivity in OCD patients. Traditional psychological treatments for OCD, like Exposure and Response Prevention (ERP), primarily target the compulsive behaviors and the obsessive thoughts. The emotional driver is addressed indirectly through habituation. This research suggests a need for a more direct and proactive focus on the dysregulated emotional state itself. In clinical practice, adjusting cognitive and emotional skills can be used as a supplement to traditional behavior centered techniques. This approach has the potential to not only improve outcomes for a broader range of patients but also to directly address the core affective vulnerability that may underlie the impulsive drive in OCD, ultimately leading to more resilient and sustainable recovery. Future research should now focus on developing and empirically testing such integrated manualized protocols.

## 5 Conclusion

In conclusion, our study found that OCD patients exhibited elevated impulsivity and tended to rely more on maladaptive rather than adaptive cognitive emotion regulation strategies. The severity of OCD symptoms, presence of depressive symptoms, and cognitive emotion regulation strategies employed all had associations with different facets of impulsivity. Notably, the severity of OCD symptoms influenced impulsivity only indirectly, through its effects on cognitive emotion regulation and depression. Since impulsivity is a significant risk factor for poorer outcomes in OCD, addressing it is clinically important. However, there is a lack of effective intervention methods for it currently. Our findings indicate that cognitive therapy targeting emotional regulation could serve as a promising approach to managing impulsivity in OCD patients.

## Data Availability

The raw data supporting the conclusions of this article will be made available by the authors, without undue reservation.
