# Supplementary material for: Impulsivity in patients with obsessive-compulsive disorder: exploring the mediating effect of cognitive emotion regulation strategies and depressive symptoms
Source: Front Psychiatry. 2025 Nov 14;16:1668538. doi: 10.3389/fpsyt.2025.1668538 (PMC12661847; doi:10.3389/fpsyt.2025.1668538)
Supplement: Supplementary file 1 [file Table1.docx]

**Model 1: Mediating effects of adaptive strategies and BDI-II score between Y-BOCS total score and BIS-11 total score in OCD patients**

Goodness of fit: *χ^2^*= 0.000, *df*=0, CMIN = 0.000，GFI=1.000, CFI=1.000, NFI=1.000，IFI =1.000，RMR =0.000 SRMR = 0.000

**Table 1.** The Paths and Effect Analysis of **A****daptive strategies→BIS-11 total score** of Model 1

| Effect | *β* | SE | 95% CI | *p* |
| --- | --- | --- | --- | --- |
| Direct effect | -0.21 | 0.13 | -0.48~0.06 | 0.113 |
| Indirect effect | -0.00 | 0.05 | -0.12~0.09 | 0.992 |
| Total effect | -0.22 | 0.12 | -0.47~0.03 | 0.086 |

**Table 2.** The Paths and Effect Analysis of **BDI-II score→BIS-11 total score** of Model 1

| Effect | *β* | SE | 95% CI | *p* |
| --- | --- | --- | --- | --- |
| Direct effect | 0.29 | 0.12 | 0.05~0.50 | 0.023^*^ |
| Indirect effect | - | - | - | - |
| Total effect | 0.29 | 0.12 | 0.05~0.50 | 0.023^*^ |

^*^ *p*< 0.05

**Non-planning impulsiveness (Y-BOCS &** **Adaptive strategies &** **BDI-II)**

Goodness of fit

*χ^2^*= 0.000, *df*=0, CMIN = 0.000，GFI=1.000, CFI=1.000, NFI=1.000，IFI =1.000，RMR =0.000 SRMR = 0.000

**Table 3.** Mediating effects of adaptive strategies and BDI-II score between Y-BOCS total score and BIS-11 non-planning impulsiveness score in OCD patients

| Path | *β* | SE | 95% CI | *p* |
| --- | --- | --- | --- | --- |
| Y-BOCS total score→BIS-11 non-planning impulsiveness score | 0.02 | 0.13 | -0.22~0.28 | 0.811 |
| Y-BOCS total score→Adaptive strategies | -0.17 | 0.14 | -0.41~0.13 | 0.255 |
| Y-BOCS total score→BDI-II score | 0.31 | 0.12 | 0.04~0.52 | 0.023^*^ |
| Adaptive strategies→BDI-II score | -0.01 | 0.16 | -0.30~0.30 | 0.977 |
| Adaptive strategies→BIS-11 non-planning impulsiveness score | -0.39 | 0.14 | -0.65~-0.10 | 0.010^*^ |
| BDI-II score→BIS-11 non-planning impulsiveness score | 0.35 | 0.12 | 0.11~0.56 | 0.004^**^ |

Note: Y-BOCS= Yale-Brown Obsessive Compulsive Scale; BDI-II= Beck Depression Inventory-II; BIS-11= Barratt Impulsiveness Scale-11. ^*^*p*< 0.05, ^**^ *p*< 0.01

**Table 4.** The Paths and Effect Analysis of mediating effects of adaptive strategies and BDI-II score between Y-BOCS total score and BIS-11 non-planning impulsiveness score in OCD patients

| Effect | *β* | SE | 95% CI | *p* |
| --- | --- | --- | --- | --- |
| Direct effect | 0.02 | 0.13 | -0.22~0.28 | 0.811 |
| Indirect effect | 0.17 | 0.06 | 0.05~0.30 | 0.010^*^ |
| Total effect | 0.20 | 0.14 | -0.09~0.44 | 0.174 |

^*^*p*< 0.05

**Attentional impulsiveness (Y-BOCS &** **Adaptive strategies &** **BDI-II)**

Goodness of fit

*χ^2^*= 0.000, *df*=0, CMIN = 0.000，GFI=1.000, CFI=1.000, NFI=1.000，IFI =1.000，RMR =0.000 SRMR = 0.000

**Table 5.** Mediating effects of adaptive strategies and BDI-II score between Y-BOCS total score and BIS-11 attentional impulsiveness score in OCD patients

| Path | *β* | SE | 95% CI | *p* |
| --- | --- | --- | --- | --- |
| Y-BOCS total score→BIS-11 attentional impulsiveness score | 0.24 | 0.11 | 0.02~0.46 | 0.039^*^ |
| Y-BOCS total score→Adaptive strategies | -0.17 | 0.14 | -0.41~0.13 | 0.255 |
| Y-BOCS total score→BDI-II score | 0.31 | 0.12 | 0.04~0.52 | 0.023^*^ |
| Adaptive strategies→BDI-II score | -0.01 | 0.16 | -0.30~0.30 | 0.977 |
| Adaptive strategies→BIS-11 attentional impulsiveness score | 0.17 | 0.12 | -0.07~0.40 | 0.163 |
| BDI-II score→BIS-11 attentional impulsiveness score | 0.31 | 0.09 | 0.12~0.46 | 0.004^**^ |

Note: Y-BOCS= Yale-Brown Obsessive Compulsive Scale; BDI-II= Beck Depression Inventory-II; BIS-11= Barratt Impulsiveness Scale-11. ^*^ *p*< 0.05; ^**^ *p*< 0.01

**Table 6.** The Paths and Effect Analysis of mediating effects of adaptive strategies and BDI-II score between Y-BOCS total score and BIS-11 attentional impulsiveness score in OCD patients

| Effect | *β* | SE | 95% CI | *p* |
| --- | --- | --- | --- | --- |
| Direct effect | 0.24 | 0.11 | 0.02~0.46 | 0.039^*^ |
| Indirect effect | 0.07 | 0.07 | -0.06~0.20 | 0.300 |
| Total effect | 0.31 | 0.11 | 0.07~0.50 | 0.015^*^ |

^*^ *p*< 0.05

**Model 2: Mediating effects of maladaptive strategies and BDI-II score between Y-BOCS total score and BIS-11 total score in OCD patients**

Goodness of fit: *χ^2^*= 0.000, *df*=0, CMIN = 0.000，GFI=1.000, CFI=1.000, NFI=1.000，IFI =1.000，RMR =0.000 SRMR = 0.000

**Table 7.** The Paths and Effect Analysis of **Maladaptive strategies→BIS-11 total score** of Model 2

| Effect | *β* | SE | 95% CI | *p* |
| --- | --- | --- | --- | --- |
| Direct effect | -0.05 | 0.15 | -0.33~0.25 | 0.689 |
| Indirect effect | 0.12 | 0.07 | 0.02~0.29 | 0.025^*^ |
| Total effect | 0.07 | 0.13 | -0.20~0.33 | 0.669 |

* *p*< 0.05

**Table 8.** The Paths and Effect Analysis of **BDI-II score→BIS-11 total** **score** of Model 2

| Effect | *β* | SE | 95% CI | *p* |
| --- | --- | --- | --- | --- |
| Direct effect | 0.31 | 0.15 | 0.00~0.57 | 0.050 |
| Indirect effect | - | - | - | - |
| Total effect | 0.31 | 0.15 | 0.00~0.57 | 0.050 |

**Non-planning impulsiveness (Y-BOCS &** **Maladaptive strategies & BDI-II)**

Goodness of fit

*χ^2^*= 0.000, *df*=0, CMIN = 0.000，GFI=1.000, CFI=1.000, NFI=1.000，IFI =1.000，RMR =0.000 SRMR = 0.000

**Table 9.** Mediating effects of maladaptive strategies and BDI-II score between Y-BOCS total score and BIS-11 non-planning impulsiveness score in OCD patients

| Path | *β* | SE | 95% CI | *p* |
| --- | --- | --- | --- | --- |
| Y-BOCS total score→BIS-11 non-planning impulsiveness score | 0.07 | 0.14 | -0.21~0.34 | 0.587 |
| Y-BOCS total score→Maladaptive strategies | 0.07 | 0.13 | -0.18~0.31 | 0.600 |
| Y-BOCS total score→BDI-II score | 0.28 | 0.11 | 0.05~0.49 | 0.016^*^ |
| Maladaptive strategies→BDI-II score | 0.38 | 0.12 | 0.13~0.58 | 0.002^*^ |
| Maladaptive strategies→BIS-11 non-planning impulsiveness score | -0.30 | 0.17 | -0.59~0.05 | 0.090 |
| BDI-II score→BIS-11 non-planning impulsiveness score | 0.48 | 0.15 | 0.16~0.74 | 0.005^**^ |

Note: Y-BOCS= Yale-Brown Obsessive Compulsive Scale; BDI-II= Beck Depression Inventory-II; BIS-11= Barratt Impulsiveness Scale-11. ^*^ *p*< 0.05; ^**^ *p*< 0.01

**Table 10.** The Paths and Effect Analysis of mediating effects of maladaptive strategies and BDI-II score between Y-BOCS total score and BIS-11 non-planning impulsiveness score in OCD patients

| Effect | *β* | SE | 95% CI | *p* |
| --- | --- | --- | --- | --- |
| Direct effect | 0.07 | 0.14 | -0.21~0.34 | 0.587 |
| Indirect effect | 0.13 | 0.07 | 0.02~0.27 | 0.022^*^ |
| Total effect | 0.20 | 0.14 | -0.09~0.44 | 0.174 |

^*^ *p*< 0.05

**Attentional impulsiveness (Y-BOCS&** **Maladaptive strategies &** **BDI-II)**

Goodness of fit

*χ^2^*= 0.000, *df*=0, CMIN = 0.000，GFI=1.000, CFI=1.000, NFI=1.000，IFI =1.000，RMR =0.000 SRMR = 0.000

**Table 11.** Mediating effects of maladaptive strategies and BDI-II score between Y-BOCS total score and BIS-11 attentional impulsiveness score in OCD patients

| Path | *β* | SE | 95% CI | *p* |
| --- | --- | --- | --- | --- |
| Y-BOCS total score→BIS-11 attentional impulsiveness score | 0.23 | 0.11 | 0.02~0.43 | 0.034^*^ |
| Y-BOCS total score→Maladaptive strategies | 0.07 | 0.13 | -0.18~0.31 | 0.600 |
| Y-BOCS total score→BDI-II score | 0.28 | 0.11 | 0.05~0.49 | 0.016^*^ |
| Maladaptive strategies→BDI-II score | 0.38 | 0.12 | 0.13~0.58 | 0.002^**^ |
| Maladaptive strategies→BIS-11 attentional impulsiveness score | 0.23 | 0.15 | -0.10~0.50 | 0.171 |
| BDI-II score→BIS-11 attentional impulsiveness score | 0.21 | 0.10 | -0.00~0.40 | 0.052 |

Note: Y-BOCS= Yale-Brown Obsessive Compulsive Scale; BDI-II= Beck Depression Inventory-II; BIS-11= Barratt Impulsiveness Scale-11. ^*^*p*< 0.05; ^**^ *p*< 0.01

**Table 12.** The Paths and Effect Analysis of mediating effects of maladaptive strategies and BDI-II score between Y-BOCS total score and BIS-11 attentional impulsiveness score in OCD patients

| Effect | *β* | SE | 95% CI | *p* |
| --- | --- | --- | --- | --- |
| Direct effect | 0.23 | 0.11 | 0.02~0.43 | 0.034^*^ |
| Indirect effect | 0.08 | 0.06 | -0.03~0.21 | 0.140 |
| Total effect | 0.31 | 0.11 | 0.07~0.50 | 0.015^*^ |

^*^ *p*< 0.05

Mediating effects of adaptive/ maladaptive strategies and BDI-II score between Y-BOCS total score and ***BIS-11 motor impulsiveness*** in OCD patients: all paths are nonsignificant.
